# Future heat adaptation and exposure among urban populations and why a prospering economy alone won’t save us

**DOI:** 10.1038/s41598-021-99757-0

**Published:** 2021-10-13

**Authors:** Linda Krummenauer, Luís Costa, Boris F. Prahl, Jürgen P. Kropp

**Affiliations:** 1 Potsdam Institute for Climate Impact Research, RD2 Climate Resilience, Potsdam, 14412 Germany; 2 Institute of Environmental Science and Geography, University of Potsdam, Potsdam, 14476 Germany

**Keywords:** Climate change, Climate-change impacts, Climate-change adaptation, Climate-change impacts, Socioeconomic scenarios

## Abstract

When inferring on the magnitude of future heat-related mortality due to climate change, human adaptation to heat should be accounted for. We model long-term changes in minimum mortality temperatures (MMT), a well-established metric denoting the lowest risk of heat-related mortality, as a function of climate change and socio-economic progress across 3820 cities. Depending on the combination of climate trajectories and socio-economic pathways evaluated, by 2100 the risk to human health is expected to decline in 60% to 80% of the cities against contemporary conditions. This is caused by an average global increase in MMTs driven by long-term human acclimatisation to future climatic conditions and economic development of countries. While our adaptation model suggests that negative effects on health from global warming can broadly be kept in check, the trade-offs are highly contingent to the scenario path and location-specific. For high-forcing climate scenarios (e.g. RCP8.5) the maintenance of uninterrupted high economic growth by 2100 is a hard requirement to increase MMTs and level-off the negative health effects from additional scenario-driven heat exposure. Choosing a 2 °C-compatible climate trajectory alleviates the dependence on fast growth, leaving room for a sustainable economy, and leads to higher reductions of mortality risk.

## Introduction

Apart from an increasing temperature trend in a warming climate, heat events will become more frequent, intense and longer lasting^1–3^. In response, human populations will have to adapt to higher future temperatures to ensure their survival^4^. The absence of adaptation to heat in urban populations would lead to an substantial increase in excess mortality in the future^5^. These new conditions will challenge human health and the habitability of some world regions^6^. Considerable efforts have been made to estimate future heat-related mortality risk^7,8^, excess mortality^5^, and future heat exposure^9–11^, or burden of heat-related mortality attributable to recent anthropogenic climate change^12^ among urban populations at a global scale. A comprehensive global projection of lethal conditions and their occurrence in the future considering possible climate futures from three Representative Concentration Pathways (RCPs) has been delivered by Mora et al.^10^. However, a better understanding of heat-related adaptation^13,14^ is required.

Studies have proposed the use of the minimum mortality temperature (MMT) as indication of human long-term adaptation to heat^15,16^. The MMT quantifies the lowest point of the temperature-mortality curve^5,8,17^ and therefore it currently is the best empirical measure of the temperature level to which a city/region is adapted to. Temperatures significantly higher or lower than the MMT come associated with disproportional increases in excess mortality. Upward changes in the MMT were found to shift the entirety of the temperature-mortality curve as well as associated indicators such as threshold values defining national heat-wave warnings^18^, hence indicating that heat adaptation is taking place^19^. In our understanding, both aspects, physiological acclimatisation and a socio-economic standard facilitating the access to technological, social or behavioural measures to avoid heat exposure are jointly considered in the MMT^20^. This has been acknowledged by separating physiological acclimatisation and non-climate driven adaptation mechanisms^21^ for observed mortality in the recent climate.

A principal method has been proposed to model the future heat-mortality relationship in cities. The exposure-response function remains in its shape and is entirely shifted to higher temperature regimes^22,23^. This method relies on a critical assumption of choosing a delta value quantifying the absolute shift of the curve. The delta value is however subject to uncertainty since it requires the knowledge of future mortality records^23^. Most authors circumvent this unknown by continuing past mortality patterns^8^.

Our research seeks to complement these previous efforts by contributing the delta value by which the MMT changes for the world’s major cities. Further, we analyse both, the populations’ adaptation to heat as the MMT, their heat exposure and the future changes in these measures. We understand a positive change in MMT as gain in adaptation. The exposure is measured as (1) frequency of MMT exceedance by the daily temperature and (2) magnitude of this exceedance. We argue that only a contextualised analysis of adaptation and exposure captures the populations’ potential to cope with heat, especially under varying climate trajectories and development pathways. A high adaptation eases the coping with high exposure, while high exposure might lead to many fatalities given a low adaptation level. Our analysis considers four possible Representative Concentration Pathways (RCPs)^24^ from four Global Circulation Models (GCMs). We combine these different climate futures with five possible socio-economic pathways (SSPs)^25,26^ leant on the Scenario Matrix^26,27^. These RCP/SSP combinations cover a broad bandwidth of possible climatic and socio-economic futures with different health outcomes for urban populations. For the first time, we offer the full spectrum of future adaptation and exposure outcomes and their future changes depending on the underlying climate trajectories and socio-economic developments. This work shows which benefits and detriments these futures have in store concerning heat-related mortality until 2100. We identify the principal influences of adaptation change for two scenarios most beneficial in economic growth but contrary in their climate trajectory. It is our objective to determine the lesser of two evils regarding the change in mortality against an ideal future in absence of climate change and heat exposure. We aim at demonstrating the disadvantage for human health arising from high forcing levels driven by a rapid growth based on fossil-fuels. Our results help to inform the public and support decision-making concerning climate and human urban health.

## Results

### General observations across cities

We assessed the changes in adaptation to heat for 3820 cities worldwide from 2000 to 2100. We considered feasible combinations of the long-term ensemble mean (ENSMEAN) of each RCP and the SSPs. The projected MMT and its 2000–2100 change, $\Delta$MMT, were contextualised with the change in two heat exposure parameters: (1) the frequency of heat exposure (EXD) and (2) the exposure magnitude (MAG). They refer to how many days the MMT is exceeded by the daily temperature in the future and in the past annually and how large the exceedance magnitude is. We used the 1991–2000 and 2090–2099 decadal means of EXD and MAG. We evaluated the parameter changes $\Delta$EXD and $\Delta$MAG between the past and the future decades (see Supplementary Table S1 for city parameters).

**Figure 1:**
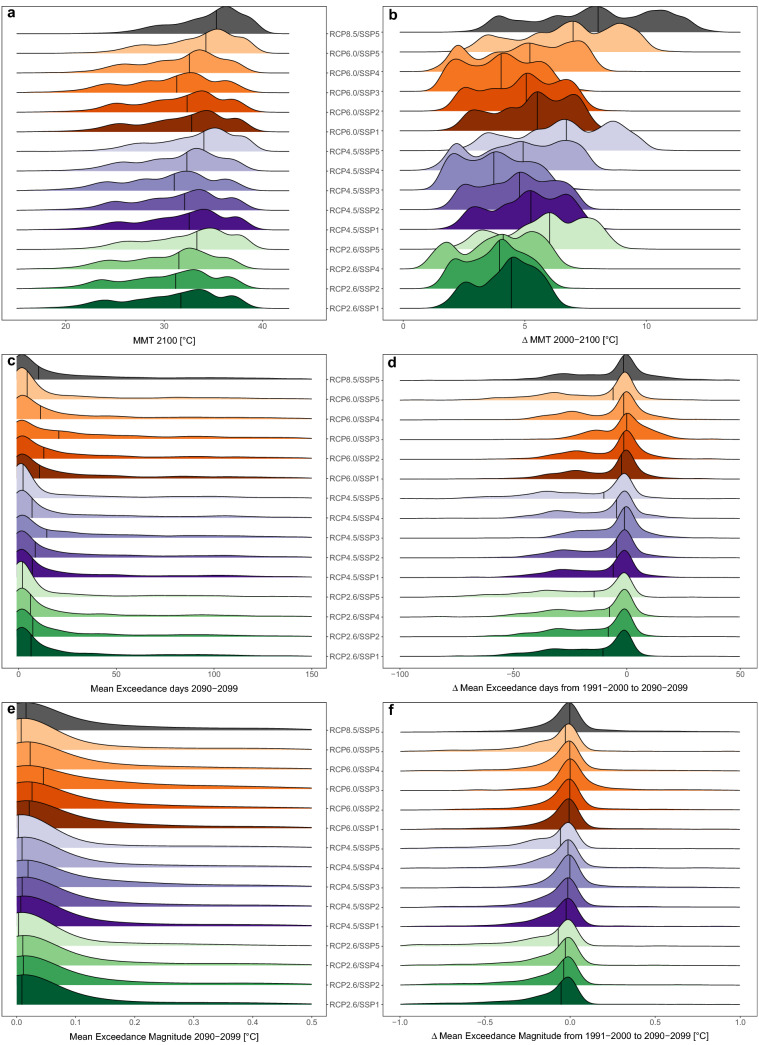
Distributions of studied adaptation and exposure parameters for the RCP/SSP combinations. Absolute MMT for 2100 (**a**), 2090–2099 exceedance days EXD (**c**), 2090–2099 exceedance magnitude MAG (**e**). Parameter changes are $\Delta$MMT 2000–2100 (**b**), and exposure from 1991–2000 to 2090–2099: $\Delta$EXD (**d**), $\Delta$MAG (**f**). Vertical lines indicate the distributions’ medians. Distributions (**c–f**) were trimmed.

MMT distributions at the end of the century varied in range and median across the RCP/SSP combinations (Fig. 1a). The distributions’ medians increase with an rising forcing level, culminating in SSP5 combinations. Combinations with SSP1 and SSP4 display second and third largest median values. Distributions associated with SSP5 and SSP1 stretch towards high MMT values. More cities reach higher adaptation in 2100 than in other combinations. The contrary applies for SSP3. SSP5 combinations, especially with RCP8.5, cover an enormous $\Delta$MMT range, while in SSP1 it is smaller (Fig. 1b). Although most distributions show low EXD, the tail towards higher EXD enlarges with increasing forcing, as obvious in RCP8.5/SSP5, and in RCP6.0 combinations (Fig. 1c). In RCP2.6 few cities show a positive $\Delta$EXD towards additional EXD in 2090–2099 but many display a negative $\Delta$EXD, a reduction in EXD (Fig. 1d). A higher forcing relates to additional EXD in more cities. RCP8.5/SSP5 exhibits a tail into larger $\Delta$EXD. Simultaneously, the number of cities experiencing reductions in EXD remains high across RCPs, notably in SSP5 and SSP1 combinations. A pattern similar to EXD is obvious in MAG (Fig. 1e). Distributions involving SSP3 have largest tails towards high MAG. The median is lowest in SSP1 and SSP5 combinations. Reductions in $\Delta$MAG are dominant in SSP1 and SSP5 combinations (Fig. 1f). Contrary, SSP3 displays highest $\Delta$MAG. The distribution of $\Delta$MAG RCP8.5/SSP5 can be distinguished by prominent tails into both $\Delta$MAG directions and by the highest median. This investigation implies, changes in adaptation and exposure across the city sample are divers, explicitly under RCP8.5/SSP5 conditions.

### How RCPs and SSP influence adaptation and exposure

**Figure 2:**
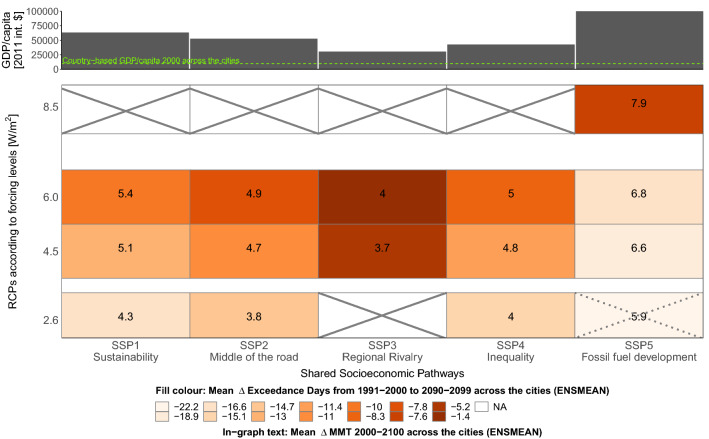
Systematic overview of the change in adaptation and exposure for the city sample according to each RCP/SSP combination and the future socio-economic level per SSP. Lower panel: The 1991–2000 to 2090–2099 $$\Delta$$EXD (orange boxes) in context of $$\Delta$$MMT (in-graph text annotations) for all possible RCP/SSP combinations. RCP2.6/SSP5 seems implausible^26^. Parameters of all scenario combinations are presented in Supplementary Table S2. Upper panel: Unique country-based GDP/capita per SSP in 2100 (mean from IIASA and OECD data). Green line in upper panel denotes the country-based GDP/capita as of 2000 [in 2011 int.$].

An investigation of mean adaptation and exposure across the cities supports the previous findings. Figure 2 summarises the effects on MMT and EXD and their changes in the cities across all feasible RCP/SSP combinations according to the Scenario Matrix^26,27^. For the city sample, the highest change in mean adaptation across all scenario combinations is a mean $\Delta$MMT of 7.9  °C, which is reached by RCP8.5/SSP5, the highest forcing level and the most rapid unsustainable economic growth. This also yields the highest absolute mean MMT for the city sample, 34.4  °C. This combination shows a small $\Delta$EXD of − 7.6 until the decade 2090–2099 and thus deviates from SSP5 combinations paired with lower forcing (Fig. 2). The lowest mean $\Delta$MMT (3.7  °C) and the lowest absolute mean MMT (30.2  °C) are displayed for RCPs 4.5 with SSP3. SSP3 is characterised by a low future socio-economic level. Combinations with RCPs 4.5 and 6.0 yield the smallest mean reductions in EXD ($\Delta$EXDs of − 5.2 and − 1.4) across the city sample until the future. A small mean $\Delta$MMT of 4.3 (and a moderate future mean MMT of 30.8  °C) across the cities is produced by RCP2.6/SSP1. This scenario combination relying on sustainable growth exhibits the second highest increment in socio-economic development until 2100. It yields a relatively large mean reduction of − 16.6 $\Delta$EXD until the future decade and equals $\Delta$EXD in RCP6.0/SSP5.

We observe that with an increasing forcing, except for SSP5 combinations, the mean $\Delta$MMT, but also the mean $\Delta$EXD become larger across the city sample. The mean $\Delta$MAG behaves likewise. It ranges between − 0.2  $^{\circ }$C  in RCP2.6/SSP1 and +0.2  $^{\circ }$C  in RCP8.5/SSP5 (Supplementary Fig. S1). Even though increasing forcing levels achieve higher adaptation and larger adaptation rates until 2100, they amplify heat exposure frequency and magnitude.

SSP5 combinations, excluding that with RCP8.5, show high socio-economic levels across the cities and perform well concerning exposure reductions and high future MMTs. Hence, a large projected GDP/capita is an advantageous precondition for high adaptation. For our city sample, SSP5 generates the highest future mean country-based GDP/capita (Int$ 101 205) compared to the beginning of the century (Int$ 9 849). The 2100 GDP/capita related to SSP1 is less, but still comparatively high (Int$ 63 346). This suggests a high GDP/capita does not automatically lead to a better bearable situation regarding future heat and its related mortality for urban populations. In the following, we contrast two scenario combinations that generate the most optimistic socio-economies and thus enable highest adaptation gains (SSP5 and SSP1) but that are contrary in future exposure (RCP8.5/SSP5 and RCP2.6/SSP1).

### Drivers of changes in adaptation 2000–2100

**Figure 3:**
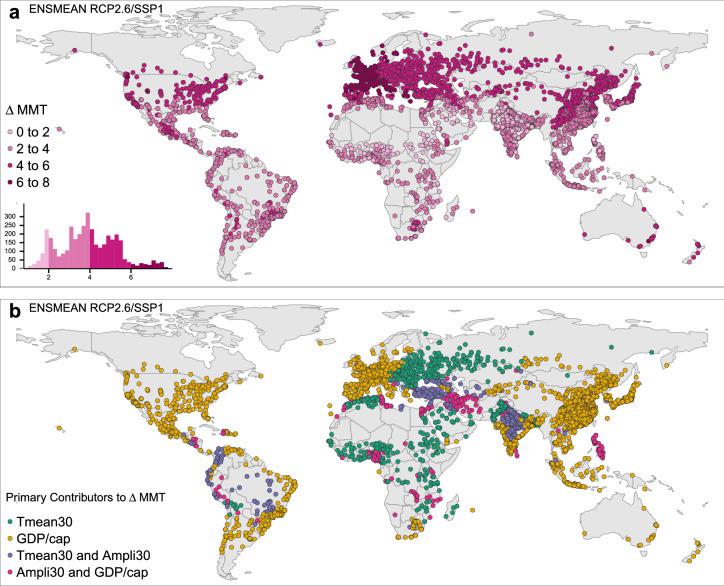
Changes in adaptation until 2100 and their primary contributors to $\Delta$MMT in a RCP2.6/SSP1 future. $\Delta$MMT 2000–2100 for RCP2.6/SSP1 for major world cities (**a**). Contributions of the single variables’ 2000–2100 changes to $\Delta$MMT 2000–2100 for RCP2.6/SSP1 (**b**). (Map created in R (version 3.6.2)^28^ using the tmap package (version 2.3-2)^29^).

**Figure 4:**
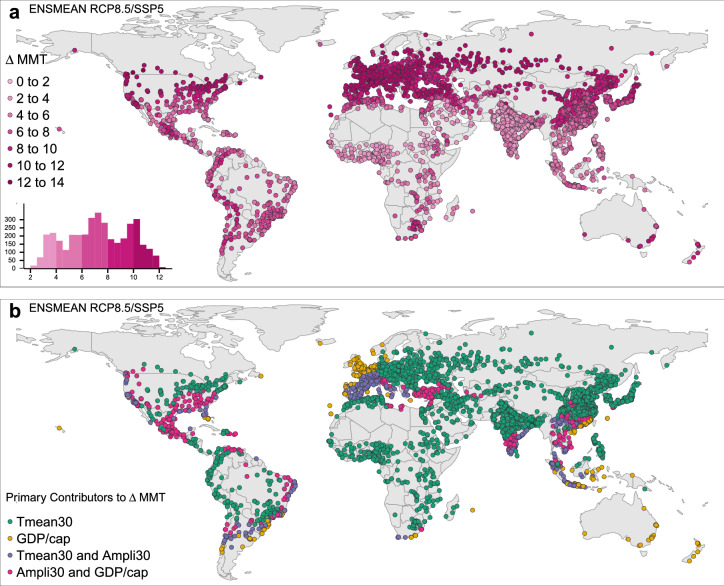
Changes in adaptation until 2100 and their primary contributors to $\Delta$MMT in a RCP8.5/SSP5 future. $\Delta$MMT 2000–2100 for RCP8.5/SSP5 for major world cities (**a**). Contributions of the single variables’ 2000–2100 changes to $\Delta$MMT 2000–2100 for RCP8.5/SSP5 (**b**). (Map created in R (version 3.6.2)^28^ using the tmap package (version 2.3-2)^29^).

An analysis of adaptation change for the scenario combinations RCP2.6/SSP1 and RCP8.5/SSP5 unveils regionally distinct change pattern of $\Delta$MMT (Figs. 3a, 4a). Cities in the northern hemisphere, especially in western Europe, show a larger $\Delta$MMT than subtropical and tropical cities or cities in the southern hemisphere, except Oceania. Evidence from the top and the bottom of the $\Delta$MMT distributions confirm these findings: Under RCP2.6/SSP1, highest $\Delta$MMTs are 7.2  $^{\circ }$C  in Ostrava and Brno (Czech Republic), 7.1  $^{\circ }$C  in Olomouc (Czech Republic), 7.0  $^{\circ }$C  in Prague and Plzen (Czech Republic) and 6.9  $^{\circ }$C  in Râmnicu Vâlcea (Romania). Lowest $\Delta$MMTs range between 1 $^{\circ }$C  (Nouakchott, Mauritania) and 1.5  $^{\circ }$C  (Umm Durman, Sudan) covering further cities in Sudan and Chad. In a RCP8.5/SSP5 future, the highest adaptation gain is a $\Delta$MMT of 13  $^{\circ }$C  in Râmnicu Vâlcea (Romania), followed by Ostrava (Czech Republic, 12.9  $^{\circ }$C ), Piatra Neamt and Olomouc (Romania and Czech Republic, 12.8 $^{\circ }$C ), and Kislovodsk and Uhta (Russia, 12.7  $^{\circ }$C ). The smallest gains in MMT range between 1.8  $^{\circ }$C  (Nouakchott, Mauritania) and 2.5  $^{\circ }$C  (Umm Durman, Sudan) and cover further cities in Sudan and Chad.

To shed light on the principal drivers behind $\Delta$MMT across cities around the globe, we calculated the changes in each variable (climate variables and GDP/capita) until 2100 and their respective isolated effects on $\Delta$MMT. The greatest weighted variable change was identified for each city and compared to the aggregated weight of the remaining variables’ changes. The resulting primary contributors to $\Delta$MMT, either a single variable’s influence or the sum of two, are illustrated in Fig. 3b for RCP2.6/SSP1 and Fig. 4b for RCP8.5/SSP5. In the former case, large changes in adaptation are solely driven by high gains in GDP/capita cities in Western Europe, North America, East Asia, Oceania and coastal South America (Fig. 3b). The change in climate as a primary driver leads to moderate increments in $\Delta$MMT in Eastern European cities. Depending on the increment in variable change, this can also result in low $\Delta$MMT, as in cities in Northern Africa, the Middle East and coastal Nigeria. This concerns cities in the Sahel, the Arabian Peninsula, and Pakistan and India, where climate-driven $\Delta$MMTs range between 0 and 2 $^{\circ }$C . Thus, the adaptation gain is lowest and slowest until 2100.

RCP8.5/SSP5 portrays higher increments in $\Delta$MMT than RCP2.6/SSP1, while the regional distribution of change pattern roughly remains similar (Figs. 3a, 4a). However, completely different variable effects dominate $\Delta$MMT in RCP8.5/SSP5 (Fig. 4b). The extensive effect of GDP/capita on high $\Delta$MMTs is either masked by an even larger climate influence or conjoined by the climate effect. Few cities in northwestern Europe, in China and in the southernmost latitudes remain whose large $\Delta$MMT is uniquely defined by socio-economic gains until 2100. In RCP8.5/SSP5 the changes in climate variables until 2100 act as primary contributors to $\Delta$MMT. Highest $\Delta$MMTs across Europe are driven by the 30-year mean temperature in the east and additionally by the 30-year mean amplitude in southern Europe. In RCP8.5/SSP5, the 30-year mean temperature dominates the $\Delta$MMT in a larger share of cities. Across African cities, Tmean30 is associated with rather low $\Delta$MMTs.

Especially in a fossil-fuel-based future with rapid socio-economic growth as in RCP8.5/SSP5, the adaptation to heat will largely be driven by a strong physiological acclimatisation until 2100 and outweigh the already strong effect of economic growth. In a sustainable prosperous future, the gain in wealth until 2100 is the primary contributor to achieve heat adaptation.

### Changes in exposure

Comparing the two highlighted scenario combinations regarding changes in exposure parameters until 2090–2099 across the cities reveals distinguished characteristics for $\Delta$EXD and $\Delta$MAG. In RCP2.6/SSP1, EXD reductions until 2090–2099 are largest with − 140 EXD in the Chilean city Antofagasta, in Mossoró (Brazil, $\Delta$EXD − 128), Coquimbo and La Serena (Chile) with $\Delta$EXD of − 98 EXD. The maximum increase in EXD is projected for Jhang Maghiana (Pakistan, $\Delta$EXD + 40). Further Pakistani cities follow (Gojra $\Delta$EXD + 38, Faisalabad and Jaranwala, Sahiwal, and Okara $\Delta$EXD + 3 7). RCP8.5/SSP5 yields more extreme EXD changes than the sustainable scenario. Still, its mean $\Delta$EXD across the cities remain twice as high (Figs. 1d, 2). The largest $\Delta$EXDs reductions are − 185 EXD in Antofagasta (Chile), − 149 EXD in Coquimbo and La Serena (Chile). Mossoró (Brasil), Copiapó (Chile), Downey (USA) follow with − 116, − 109 and − 104 EXD. The maximum EXD increase for this scenario is a $\Delta$EXD of + 92 EXD in Ciudad Obregón (Mexico), which exceeds the maximum increase in RCP2.6/SSP1 by factor 2.3. Further Pakistani cities rank high in $\Delta$EXD: Faisalabad and Jaranwala ($\Delta$EXD + 86), Sahiwal, Okara and Bahawalnagar ($\Delta$EXD + 82).

**Figure 5:**
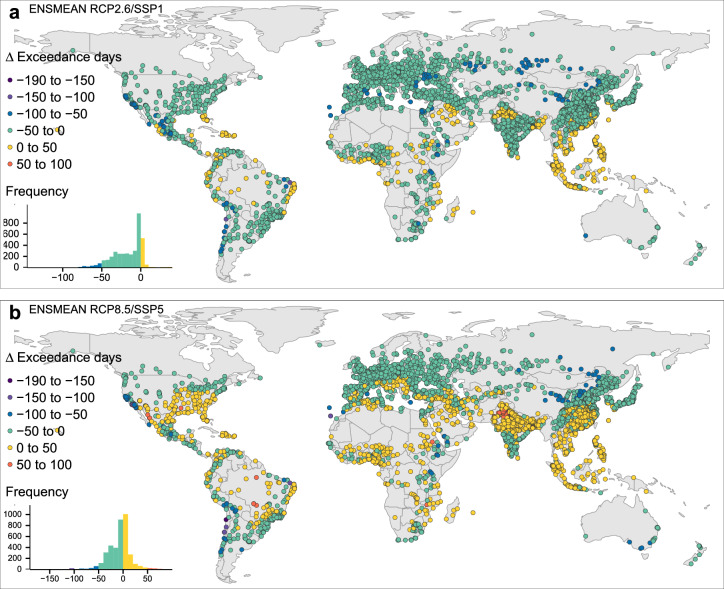
Changes in future heat exposure frequency in major cities worldwide until 2100. $\Delta$EXD 1991–2000 to 2090–2099 in case of RCP2.6/SSP1 (**a**) and in case of RCP8.5/SSP5 (**b**). (Map created in R (version 3.6.2)^28^ using the tmap package (version 2.3-2)^29^).

The global perspective on $\Delta$EXD shows prominent differences between the two highlighted scenario combinations in southern European, North American, Subsaharan, Indian, and East Asian cities, (Fig. 5). Here, in RCP2.6/SSP1, the negative $\Delta$EXDs cause reduced future EXD (− 50 to 0 EXD) (Fig. 5a). In RCP8.5/SSP5, the positive $\Delta$EXDs will yield additional EXDs (0–50 EXD) in those cities until 2090–2099 (Fig. 5b). Large scenario disparities are obvious in cities in northwestern Mexico, Brazil, Pakistan, and India. In a fossil-fuel dependent future, these cities will have to cope with 50 to 92 additional EXD until 2090–2099. In RCP2.6/SSP1 $\Delta$EXD will be less in and even bring EXD reductions in some of these cities (− 50 to 0 EXD).

**Figure 6:**
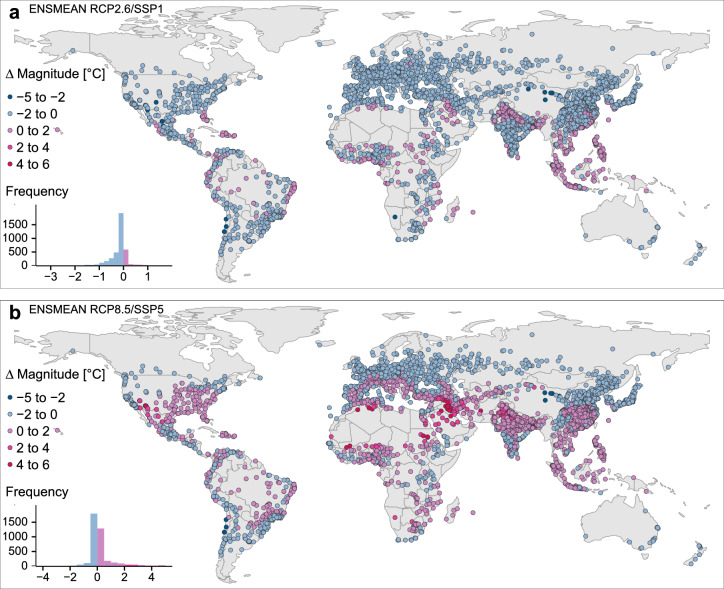
Changes in future heat exposure magnitude in major cities worldwide until 2100. $\Delta$MAG 1991–2000 to 2090–2099 in case of RCP2.6/SSP1 (**a**) and in case of RCP8.5/SSP5 (**b**). (Map created in R (version 3.6.2)^28^ using the tmap package (version 2.3-2)^29^).

In terms of $\Delta$MAG in the sustainable scenario, the largest MAG decreases concern Copiapó (Chile) with a $\Delta$MAG of − 3.5 $^{\circ }$C , Dunhuang (China, $\Delta$MAG − 3.2 $^{\circ }$C ), Coquimbo and La Serena (Chile, $\Delta$MAG − 3 $^{\circ }$C ), Geermu (China, $\Delta$MAG − 2.9 $^{\circ }$C ). The maximum increments in $\Delta$MAG are expected in Pakistan (Fig. 6a): Chishtian Mandi ($\Delta$MAG + 1.9 $^{\circ }$C ), Sahiwal, Okara, Bahawalnagar and Kamalia ($\Delta$MAG + 1.8 $^{\circ }$C ), and Gojra ($\Delta$MAG + 1.7 $^{\circ }$C ). Some of these cities overlap with highest ranks in $\Delta$EXD. Generally, for RCP2.6/SSP1 we record a $\Delta$MAG between − 2 and 0 $^{\circ }$C  for most cities. Some cities in the subtropics and tropics display a positive $\Delta$MAG between 0 and 2 $^{\circ }$C  until the future decade (Fig. 6a). MAG reductions in RCP8.5/SSP5 are more extreme compared to the sustainable future. The mean of the RCP8.5/SSP5 $\Delta$MAG across the cities is still higher and positive (Supplementary Fig. S1). Maximum MAG reductions are projected in Copiapó and Antofagasta (Chile, $\Delta$MAG − 4.4 $^{\circ }$C  and − 3.9 $^{\circ }$C , Geermu (China, $\Delta$MAG − 3.8 $^{\circ }$C ), Coquimbo and La Serena (Chile, $\Delta$MAG − 3.5 $^{\circ }$C ) and Dunhuang (China, $\Delta$MAG − 3.2 $^{\circ }$C ). Maximum $\Delta$MAG increments are observed in Bechar (Algeria, $\Delta$MAG + 5.4 $^{\circ }$C ), Karbala, al-Fallujah (Iraq) and Zambol (Iran) ($\Delta$MAG +5 $^{\circ }$C ), ar-Ramadi and as-Samawah (Iraq, $\Delta$MAG + 4.8 $^{\circ }$C ). RCP8.5/SSP5 conveys an intensified situation concerning the extremes, while $\Delta$MAG is still small in many cities (Fig. 6b). Mainly cities in the Sahel, the Middle East into Pakistan and India, in northern and southern Africa, in the Southwestern USA and in northern Mexico show high increments in $\Delta$MAG (+ 2 $^{\circ }$C  to +6 $^{\circ }$C ) until 2090–2099. Some severely exposed cities would profit from MAG reductions in RCP2.6/SSP1 instead (Fig. 6a,b).

Further evidence sharpens the disparity in future exposure concerning the selected scenario combinations (Table 1). In a RCP8.5/SSP5 future, a total of 2285 (60%) cities in our sample will profit from an EXD reduction and 1968 (51%) from a MAG reduction. Still, 1150 (30%) cities will experience additional EXD and 1459 (38%) a larger MAG. No EXD or MAG changes will concern 385 (10%) and 393 (10%) cities cities. In our sample, 705 (18%) cities will face more than one fourth of the year being EXD and 185 (5%) cities half the year being EXD. For five cities we project almost the entire year to be EXD (> 350 EXD) considering such future. RCP2.6/SSP1 in contrast is associated with less cities experiencing aggravated exposure changes until 2090–2099. Only 224 (6%) cities will face additional EXD and 338 (9%) a higher MAG. The majority of cities in 2090–2099 will profit from less EXD (3207 cities, 84%) and from a lower MAG (3074 cities, 80%). No changes in EXD or MAG will affect 389 (10%) and 408 (11%) cities. These findings imply that a lower forcing yields a larger reducing effect on the heat exposure parameters for more cities due to a milder climate change. This suggests, in an ideal future without climate change but high wealth-driven adaptation, exposure measures would be minimised and a massive reduction in mortality could be expected. Against this ideal future, RCP2.6/SSP1 constitutes a slight impairment leading to a higher mortality. RCP8.5/SSP5 signifies a substantial worsening of prospects because positive wealth effects on adaptation are likely annihilated.

**Table 1:** Number and share of cities affected by changes in exposure from 1991–2000 to 2090–2099. Exposure outcomes by indicator across the 3820 world cities comparing RCP2.6/SSP1 and RCP8.5/SSP5.

Indicator	RCP2.6/SSP1		RCP8.5/SSP5	
	No. of cities	Percent (%)	No. of cities	Percent (%)
Days reduction in EXD	3207	84	2285	59.8
Days increase in EXD	224	5.9	1150	30.1
No change in EXD	389	10.2	385	10.1
>25% of the year EXD	527	13.8	705	18.5
>50% of the year EXD	146	3.8	185	4.8
>350 EXD	0	0	5	0.1
Reduction in MAG [°C]	3074	80.5	1968	51.5
Increase in MAG [°C]	338	8.8	1459	38.2
No change in MAG	408	10.7	393	10.3

### Adaptation and heat exposure in context

**Figure 7:**
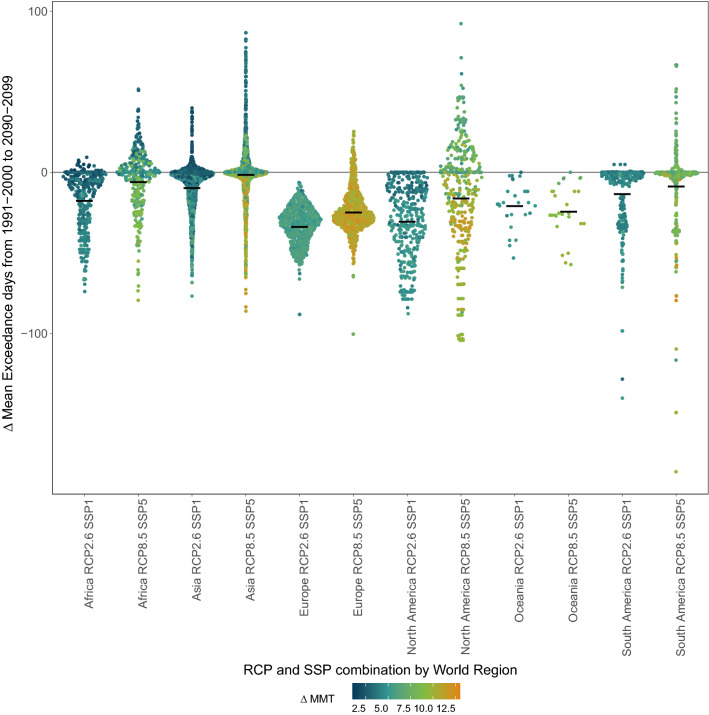
Regional disparities in $\Delta$MMT 2100–2000 and $\Delta$EXD 1991–2000 to 2090–2099 for the selected scenario combinations RCP2.6 paired with SSP1 and RCP8.5 paired with SSP5. Midbar indicates the mean of the $\Delta$EXD distribution across the sample cities.

It is indispensable to view adaptation and exposure jointly. We aggregated the adaptation and exposure parameters across our sample of 3 820 cities and provide their outcomes (P05, P95, Mean, Min and Max) in Supplementary Table S2. By 2100, a higher adaptation can be achieved by RCP8.5/SSP5 compared to RCP2.6/SSP1 because the $\Delta$MMT increment is greater. This also requires a faster adaptation rate until 2100. However, a future according to RCP2.6/SSP1 is able to minimise heat exposure for our city sample in contrast to RCP8.5/SSP5. In both scenarios $\Delta$EXD and $\Delta$MAG values mostly correlate as in cities in the Middle East, Pakistan and in parts of the USA and northern Mexico. High increases in heat exposure also coincide with small $\Delta$MMTs in these regions (Fig. 7). Such circumstances are obvious in Asian cities at the tip of the distribution in RCP2.6/SSP1, and in Asian and African cities in RCP8.5/SSP5 (Fig. 7). This suggests these cities might be prone to increases in mortality until 2100.

## Discussion

For the first time, future adaptation and heat exposure for 3820 cities worldwide have been assessed jointly. For the full spectrum of possible future climate trajectories (RCPs 2.6, 4.5, 6.0 and 8.5) and socio-economic pathways (SSPs 1–5) this analysis envisages potential adaptation and exposure changes until 2100. Considering that all options constitute an impairment against an ideal future assuming no climate change and thus only minimal exposure, we aimed to identify the least unfavourable choice for human health outcomes. We contrasted two options of highest socio-economic development facilitating wealth-enabled adaptation but diverging heat exposures: (1) The minimum forcing RCP2.6 reduces heat exposure in the majority of cities until the future decade (2090–2099) while a stable and sustainable socio-economic pathway SSP1 will equip most world cities with a moderately high adaptation. (2) A rapid socio-economic growth based on fossil fuels along with high forcing (RCP8.5/SSP5) will reach higher adaptation levels in 2100 for most cities against a sustainable future. Though, fewer cities will profit from decreases in heat exposure. In a RCP8.5/SSP5 future, 5.1 times as many cities will face more frequent heat exposures and 4.3 times as many will experience higher exposure magnitudes than in RCP2.6/SSP1. The high socio-economic level driven by fossil-fuel exploitation can only partly compensate for its own negative consequences for human health, especially as adaptation changes are rather climate-induced than by economic growth. A future according to RCP8.5/SSP5 would critically endanger cities in arid and semi-arid climates, i.e. cities in the southwestern USA, northern Mexico, in Brazil, in the Sahel, across the Arabian Peninsula and the Middle East, in Pakistan and India. All exhibit small gains in adaptation while heat exposures are projected to increase. Likely, this will raise heat-related mortality in those cities at the end of the century compared to its beginning. Even if heat exposure cannot be minimised as in a world without climate change, RCP2.6/SSP1 in contrast to RCP8.5/SSP5, has proven to be beneficial for urban populations’ health. Its associated exposure decline until 2100 will outperform that in RCP8.5/SSP5. RCP2.6/SSP1 reaches moderate adaption levels without the expense of amplifying climate change. We conclude that socio-economic growth only contributes to future heat adaptation as long as it is generated sustainably.

The approach presented here takes stock of urban populations’ adaptation and exposure to heat in 3820 cities across the globe for the full spectrum of climate trajectories and socio-economic pathways. Recent approaches have either selectively established heat-mortality relationships for only few RCPs, falling short to provide a general overview, or they present the full RCP bandwidth^5,8^. They usually do not consider future socio-economic developments^8^. We demonstrated the necessity to integrate a socio-economic aspect to assess future adaptation. We found urban populations’ heat adaptation is not purely climate-driven. Locally-extended SSPs were employed jointly with RCPs by a single study to estimate future summer excess mortality in Greater Houston (USA)^30^. They confirm other influences related to socio-economy.

So far, mostly single regions have been studied concerning habitability or heat burden. One global study considers humid heat, concluding that subtropical coastal areas are at risk^31^. For the Middle East, future habitability was questioned based on future temperature and humidity conditions regarding RCP8.5^6,31^. Besides Middle Eastern cities, we identified further cities in warm arid and semi-arid regions to be prone to increased future heat exposure and low adaptation gains according to RCP8.5/SSP5. Following a sustainable pathway avoids exposure for most cities endangered in a RCP8.5/SSP5 future. Especially western European cities, North American, Central and Eastern Asian cities would benefit from considerable and sustainable economic growth-driven adaptation gains in a RCP2.6/SSP1 future. Sustainable and stable economic growth contributes to adaptation and maintains low forcing. Even though tropical and subtropical cities show relatively small and climate-driven increments in adaptation change until 2100, they profit from small exposure changes in a sustainable scenario. Additionally, their populations are acclimatised to high temperatures today. A strong socio-economic development based on fossil-fuel exploitation as in RCP8.5/SSP5 does not principally drive adaptation but amplifies extreme heat exposures in a larger number of cities that would not be impacted as severely in a sustainable future.

It is common to investigate solely heat exposure in form of exposure days above a certain temperature threshold, e.g. high-risk days associated with mortality^11^. Such information is not quite sufficient to identify endangered locations for two reasons: First, adaptation is not accounted for, and second, because metrics describing adaptation and exposure changes matter rather than absolute values. Thus, our contextual assessment of future adaptation and exposure and most essentially their changes until the end of the century, is a novelty. We deliver a critical unknown required to determine the future HMR: $\Delta$MMT is the denominator in the fraction to calculate the slope of the future HMR, which was up to date matter to uncertainty^22,23^. Having shed light on this previously unknown delta value is a major achievement of our work.

The knowledge of the changes in heat exposure frequency and the exposure magnitude for a location leads us to conclude that a functional relationship between these parameters can be interpreted as a proxy for the change in heat-related mortality for this location. We propose a simple functional relation as a proxy for change in future heat-related mortality: $\Delta$MORT = f($\Delta$EXD, $\Delta$MAG). $\Delta$MORT is another critical metric still missing to derive the future HMR’s slope. Given the present mortality of a location and assuming no changes in demography and health status, the change in mortality until 2100 can be roughly approached through the here introduced functional relationship between the changes in exposure parameters. Approaching the future HMR through a linear method, $\Delta$MORT fills in the numerator of the slope fraction of the future HMR, which can now be solved: m = $\Delta$MMT/$\Delta$MORT. We encourage further research on this idea. Previous studies tried to circumvent the slope-related uncertainty in mortality changes by simply continuing past mortality pattern, which grounds on many assumptions^8^.

Our study has some limitations. Since to our knowledge there is no definite evidence about the time adaptation requires, we carry out our analysis under the premise that adaptation to heat, especially the wealth-enabled share, is instantaneous. We attempt to account for a lag in physiological acclimatisation by using mean climate variables over a 30-year period ending in 2094. Accordingly, our results denote a lower boundary of a wide adaptation margin. We assume no changes in health condition and heat-related mortality among the city populations until 2100. Further, the SSPs rely on assumptions and could be source of uncertainty in our projections. Still, the SSPs originate from an established framework developed to analyse different climate and socio-economic futures. We focused on ensemble means instead of scrutinising the influence of GCMs on our results. We are confident to present this information as an inter-model comparison in the future.

Urban populations will be able to adapt to increasing temperatures majorly in two ways. Either by a high socio-economic standard in a future related to sustainable economic growth. Or unfavourably, being forced to acclimatise to elevated heat exposure driven by high forcing which likely results in increased heat-related mortality in cities. We demonstrate the indispensability to appraise heat adaptation and exposure jointly to give recommendations for the public and decision-makers. We conclude that it is most beneficial to strive for a sustainable but prosperous future, which is compatible with the 2 $^{\circ }$C  limit, as for instance the scenario variant RCP2.6/SSP1 to avoid additional future heat exposure. The majority of the world’s city population will profit from such choice. National health systems, particularly in poorer regions will not be as challenged as in less sustainable high-forcing futures.

## Methods

### Climate data

*Historical modelled data* Historical climate data from the Coupled Model Intercomparison Project 5 (CMIP5) experiments^32^ were available from the Inter-Sectoral Impact Model Intercomparison Project (ISIMIP)^33^ for four General Circulation Models (GCMs): GFDL_ESM2M, HadGEM2_ES, IPSL_CM5A-LR, and MIROC5. The gridded data were obtained with spatial resolution of 0.5° × 0.5° and the ensemble mean (ENSMEAN) across the four GCMs was calculated. *Climate projections* Climate projections for four greenhouse gas concentration trajectories, the Representative Concentration Pathways (RCPS 2.6, 4.5, 6.0, and 8.5) were available from the same source as the historical modelled data. Data representing each RCP had been generated from four GCMs: GFDL_ESM2M, HadGEM2_ES, IPSL_CM5A-LR, and MIROC5. We calculated the ENSMEAN across the GCMs. The climate projections from 2006 to 2099 were provided as gridded data with a spatial resolution of 0.5° × 0.5°. *Climate variables* We used the daily mean temperature (tas) of each GCM for all four RCPs to derive the required input climate variables for each city. The 30-year mean of the daily temperature and the annual amplitude were calculated for the past period 1965–1995 as input variables to estimate MMTs for 2000. The two climate variables were calculated for the future period 2064–2094 as input variables to estimate MMTs for 2099. The latter variable was calculated from the average of each annual amplitude between the warmest and the coldest monthly mean temperature for each year within the 30-year period. The daily mean apparent temperature (AT) was derived for 2000 and for the climate prediction year 2099. This enables to assess the frequency of days which show a daily ATmean exceeding the MMT in each city, and to measure the magnitude of the MMT exceedance. We use the climate parameters daily mean temperature (tas) and the daily mean humidity (hurs) to generate the daily mean dew point temperatures (tdewp) for the prediction years 2000, and 2099 as well as for ten consecutive years in 2090–2099 employing Eq. (1)^34^. We proceed likewise with ten consecutive years in 1991–2000.

1 $$\begin{aligned} TD = B_l [ln(RH/100) + (A_l \times T)/(B_l+T)]/A_l - ln(RH/100) - (A_l \times T)/(B_l+T), \end{aligned}$$

where $$A_l=$$ 7.625 and $$B_l=$$ 243.04 $$^{\circ }$$C.

We subsequently use tas and the newly derived tdewp to calculate the daily ATmean according to the Eq. (2) by Michelozzi (2007)^35^

2 $$\begin{aligned} AT = - 2.653 - (0.994 \times T)+(0.0153 \times TD), \end{aligned}$$

where T is the daily mean temperature and TD is the daily mean dew point temperature. We correct the AT for temperatures above 34 $^{\circ }$C  for wind speed using the climate variable daily mean wind speed (sfcWind)^36^.

### Socio-economic data

Historical GDP/capita (2011 int.$). This data was given in purchasing power parity (PPP)^37^ at country level and the data on improved urban water sources (% population with access)^38^ were obtained from the World Bank Open Database. We used the data for the year 2000. Projections for the GDP/capita (2005 int.$, in PPP). Data was available at the country level representing five distinct Socio-economic Pathways (SSPs 1 to 5) from two modelling groups, IIASA^39^ and OECD^40^. The data for each SSP was adjusted to the historical GDP/capita (given in 2011 int.$) using the common base year 2015 available in both, historic and projected data. Dividing the SSP data by the quotient of the SSP data and the historic data, as an adjustment factor, levelled the projected GDP/capita to the 2011 int. Dollar. We use the 2100 mean of the two SSP datasets to estimate the MMT in 2100.

### Topographic data

Elevation data was obtained from a SRTM 90m DEM^41^ at a spatial resolution of 0.25° × 0.25°. This data was aggregated to match the resolution of the climate data at 0.5° × 0.5°.

### City coordinates

City coordinates were available from the from the Global Rural–Urban Mapping Project (GRUMP) Settlement Points^42^ from the Center or International Earth Science Information Network (CIESIN).

### Model calibration

The methodology to estimate the future MMT grounds on our previously established generalised multivariate non-linear model to approximate the MMT for cities published in Krummenauer et al.^20^. Our approach estimates the MMT for cities independently from daily mortality records on the basis of a set of city-specific climatic, topographic and socio-economic variables^20^. This allows for spatially and temporally flexible model application. Details on the previous model, how it was established in our previous study as well as on its advantages and limitations are provided in the Supplementary Methods and Discussion. The model to determine MMTs established in our previous study^20^ was adjusted for the use with the gridded historic and projected climate data from the ISIMIP project. For model calibration, we use the ENSMEAN historic 30-year mean of the daily temperature and the ENSMEAN 30-year mean of the annual amplitude calculated from the historic gridded data for the reference periods in accordance with the original work listed in the previous study^20^. The socio-economic variables for the year 2000 and the topography remained the same as in the previous study. The final selection of independent variables used in this paper, the treatment of co-linearity and the significance testing is discussed at lengths in Krummenauer et al.^20^. A total of 13 independent variables reflecting the topography (e.g. distance to coast, latitude) and socio-economy (e.g. health expenditure, share of population above 65) were originally tested in their ability in reproducing the observed MMT of the city. The same 400 cities across all climate zones and world regions were used for model calibration as in Krummenauer et al.^20^. We used a non-linear-least-squares fit to re-calibrate the model. The newly derived coefficients were then employed for MMT estimation in the historic and the future periods. We found that the re-calibrated model using gridded climate data preserves the relative importance of all independent variables as in Krummenauer et al.^20^ (see Supplementary Table S3 of the Supplementary Methods and Discussion). The RMSE of the re-calibrated model ranked at 3.2 $^{\circ}$ C while the RMSE of the original model was 2.8 $^{\circ}$ C, this indicates only a minor penalty of using coarser gridded climate data.

### Estimation of future minimum mortality temperatures

First, the newly calibrated model and the new coefficients were used to estimate the MMTs for the historic situation in 2000, employing the computed ENSMEAN long-term climate variables for 1965–1995 for each RCP, the socio-economic variables for 2000 and the topography. Second, the MMTs for the future situation in 2100 were estimated employing the computed ENSMEAN long-term climate variables for each RCP for 2065–2095, and the future socio-economic variable GDP/capita projecting the situation at the end of the century as in each SSP. The variable access to water and the topography remained constant. The future MMT estimation was carried out for each of the 15 feasible RCP and SSP combinations according to the Scenario Matrix^26,27^. This excludes the combination RCP 2.6. with SSP3 and RCP 8.5 may only be paired with SSP5. The feasibility of combination RCP2.6 and SSP5 is questionable in a real world^26^ and thus is rather neglected in our study. The MMTs are valid for the years around the prediction years 2000 and 2100 since long-term physiological acclimatisation is accounted for by employing long-term climate variables. Adaptation likely does not change significantly from one year to another. We determine the delta change in MMTs ($\Delta$MMT) between the end and the beginning of the century for each city. We carry out this delta MMT calculation on the basis of the historic MMTs for the cities and the 15 sets of projected MMTs for the cities, each for one of the RCP/SSP combinations. Subsequently, we derive the mean MMT and the mean $\Delta$MMT across the 3 820 cities for each of the RCP/SSP combinations. All calculations have been performed in R (version 3.6.2)^28^, maps have been produced with the tmap package (version 2.3-2)^29^.

### Exposure calculation

We use the estimated MMTs, a measure for human heat adaptation in cities, as a threshold to determine critical heat exposure that exceeds the MMTs and thus also the long-term acquired heat adaptation among the city population. We count the annual number of days that show a daily mean apparent temperature (ATmean) exceeding the MMT as adaptation measure. We do so for each year and city within the historic exposure reference period 1991–2000 and use the city-specific MMTs that are valid around the year 2000 as threshold. We calculate the mean number of exposure days (EXD) over the historic decade for each city. We repeat the procedure likewise for each year within the future exposure period 2090–2099 and each city under each of the four RCPs. For the future evaluation of EXD we employ the respective city-specific MMTs as thresholds that are valid around the year 2100. The choice of MMT sets that could be used as thresholds was determined by the RCP setting of the future decadal climate data. The RCP setting of the MMT sets (across the SSP settings) had to match that of the daily climate data within the decade. For example, four MMT sets for RCP2.6, combined with either SSP1, SSP2, SSP4 or SSP5 served one after another as threshold set. For the EXD analysis under RCP8.5 only MMTs for RCP8.5/SSP5 could be used. Subsequently, the mean number of exceedance days across the future decade is computed for each city and for each RCP/SSP combination. The EXD analysis resulted in 15 datasets, one for each RCP/SSP setting, covering 3 820 city-specific mean EXD values. We derive the delta changes in EXD ($\Delta$EXD) for each city by subtracting the city-specific decadal mean EXD in 1991–2000 from the city-specific mean EXD in 2090–2099 under the 15 different RCP/SSP setups. This resulted in 15 different datasets, one for each RCP/SSP setup, each containing the $\Delta$EXD for 3820 cities. During the EXD evaluation, we record the exceedance magnitude as the difference between the MMT and the daily ATmean in each city and for each year within the historic and future decades. The annual mean exceedance magnitude (MAG) is computed for each city for the 15 RCP/SSP settings. The delta change in mean MAG ($\Delta$MAG) for each city is derived by subtraction of the city-specific decadal mean MAG in 1991–2000 from the city-specific mean MAG in 2090–2099 under the 15 different RCP/SSP setups. Correspondingly, this resulted in 15 datasets containing the $\Delta$MAG for 3820 cities. Finally, we compute the RCP/SSP combination-specific mean values across the 3820 cities for each of the four exposure parameters. All calculations have been performed in R (version 3.6.2)^28^, maps have been produced with the tmap package (version 2.3-2)^29^.

## Supplementary Information

Supplementary Information.

Supplementary Table S1.

Supplementary Table S2.
