# Supplementary material for: Future heat adaptation and exposure among urban populations and why a prospering economy alone won’t save us
Source: Sci Rep. 2021 Oct 13;11:20309. doi: 10.1038/s41598-021-99757-0 (PMC8514539; doi:10.1038/s41598-021-99757-0)
Supplement: Supplementary file 1 — Supplementary Information. [file 41598_2021_99757_MOESM1_ESM.pdf]

Supplementary information: Future heat adaptation and exposure among urban populations and why a prospering economy alone won't save us

Linda Krummenauer[1,2*], **Luís Costa**[1+], **Boris F. Prahl**[1+], **and Jürgen P. Kropp**[1,2]

[1]Potsdam Institute for Climate Impact Research, RD2 Climate Resilience, Potsdam, 14412, Germany
[2]University of Potsdam, Institute of Environmental Science and Geography, Potsdam, 14476, Germany
[*]lindakrummenauer@pik-potsdam.de
[+]these authors contributed equally to this work

Supplementary Figures

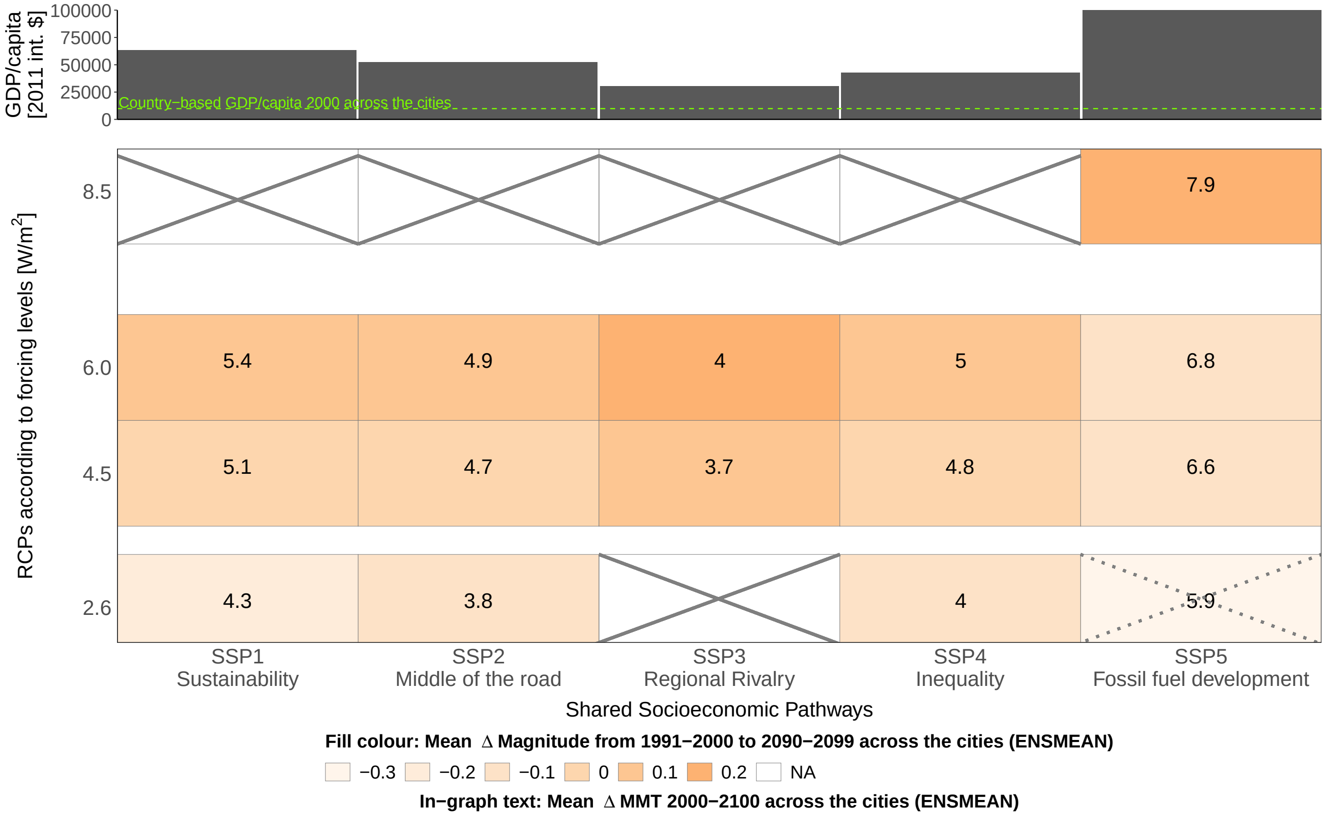

Supplementary Figure S1. Systematic overview of the change in adaptation and exposure for the city sample according to each RCP/SSP combination and the future socio-economic level per SSP. Lower panel: The 1991–2000 to 2090–2099 ΔMAG (orange boxes) in context of ΔMMT (in-graph text annotations) for all possible RCP/SSP combinations. RCP2.6/SSP5 seems implausible[1]. Parameters of all scenario combinations are presented in Supplementary Table S2. Upper panel: Unique country-based GDP/capita per SSP in 2100 (mean from IIASA and OECD data). Green line in upper panel denotes the country-based GDP/capita as of 2000 [in 2011 int.$].

Supplementary Tables

Supplementary Table S1. City-specific outcomes for the ENSMEAN values of MMT (2100), and the mean exposure parameters EXD and MAG over the decade 2090–2099, and the parameter changes DELTA.MMT (2000–2100), and the 1991–2000 to 2090-2099 decadal means of DELTA.EXD and DELTA.MAG for 15 possible RCP/SSP combinations referred to in this research.
`Supplementary_Table_S1_city_data_ENSMEAN_2100_and_2090_2099_mmt_exd_mag_dlt_values_DECADE_20210820.xlsx`

Supplementary Table S2. Descriptive statistics (P05, P95, Mean, Min, Max) of historic and projected ENSMEAN adaptation and exposure parameters and their changes until the end of the century for 15 RCP/SSP combinations referred to in this research: historic and projected MMT (MMT 2000, MMT 2100), and the historic and projected exposure parameters EXD and MAG, referred to as EXD 2000 and EXD 2100, MAG 2000 and MAG 2100 (note: exposure parameters correspond to the decadal means 1991–2000 (historic) and 2090–2099 (projected)), and their parameter changes MMT Delta (corresponds to the 2000–2100 change), and exposure parameter changes EXD Delta, MAG Delta (note: change in exposure parameters correspond to the change in decadal means from 1991–2000 to 2090–2099))
`Supplementary_Table_S2_compare_meanENSMEAN_2000_2100_and_1991_2000_to_2090_2099_dlt_mmt_exd_mag_DECADE_20210820.xlsx`

Supplementary Methods and Discussion

Methods previously used

Details on the previously developed MMT model

The model established in our prior study[2] approximates the MMT for cities without relying on daily mortality records as conventional studies do. It uses a set of city-specific climatic, topographic and socio-economic data instead. The model was systematically developed under the premise of simplicity and robustness, testing a multitude of model candidates containing different combinations of independent variables, which were gathered from previous literature. Collinearity among variables was restricted and for each model candidate individually, the significance of each model parameter was assessed via the likelihood-ratio test (LRT) with a significance level of 0.99. Model candidates that returned insignificant parameters according to our condition were removed. A further model selection criterion was the Akaike information criterion corrected for small sample sizes (AICc), which allows the comparison of non-nested models. The model candidate ranking lowest in the AICc was chosen. The model selection according to the combination of LRT and AICc consistently dismissed model candidates containing uninformative parameters while only exhibiting small increases in AICc scores (AICc difference < 2) compared to other model candidates without redundant parameters. We generally compared systematically the same variable setups in a linear model variant, segmented model variants with asymptotes and a sigmoid model variant. The latter showed the lowest and most optimal AICc of 1782 and a low RMSE of 2.81 ($^\circ$C) when employing five significant city-specific variables: the 30-year average of the daily mean temperature, the 30-year average of the annual amplitude, the elevation, the GPD/capita and improved urban water access. The other model variants showed less optimal AICc and RMSE values. The model was trained on 360 MMTs for cities across the globe and validated on 40 urban MMTs, the latter returned an RMSE of 2.63 ($^\circ$C). We did not find any systematic bias in any estimation subset for different climate zones and different world regions underlining the applicability of the model for cities across the globe. The performance of our model in estimating MMTs was better than using the most optimal temperature percentile, the 89th percentile in our daily mean temperature dataset, as suggested by previous studies. We used our model to estimate the MMT for current climate conditions for 600 European cities finding a pronounced decline in MMTs from southern European and Mediterranean cities to northern European cities, with the exception of cities in higher altitudes. The maximum MMT was 27.8°C in Sevilla (Spain).

Discussion of previous method

Advantages of the previous approach

A major advantage of our model is that it estimates MMTs as a measure of human heat adaptation independently from daily mortality records. In contrast to conventional studies analysing the heat-mortality relationship for cities based on daily mortality records, our method is in a twofold way a very flexible tool because it relies on freely available and robust, less error-prone city-specific data. First, the model employment is spatially flexible. It can be applied to any city around the globe and inform about the city-specific heat adaptation without having been subject to research using the conventional approach to derive

MMTs on the basis of daily mortality records. The spatial appliance of the model has been successfully proven in our previous study on 600 European cities. The model performance was equally robust across different climate zones and world regions. Second, our model can be adjusted for usage for different time frames. This is due to the nature of the variables in the model. While topographic input data remains the same, for climate and socio-economic input variables, projections of climate and socio-economy can be used. Thus, the MMT for future time frames can be approached with our model. It even allows to compare MMTs for different time periods and calculate the delta changes in MMT. Such time independence cannot be achieved by the conventional method to derive the MMT, where usually observed mortality time series are replicated and continued into the future. Another principal advantage of our approach is that it allows to separate the shares of physiological acclimatisation and wealth-enabled measures to overall heat adaptation. The MMT is therefore more than a simple temperature index.

Limitations of the previous approach

We have to acknowledge that some degree of uncertainty in our model had been brought about by the original MMTs from the studies used in the previous work. It has to be noted that the MMTs gathered from the studies are commonly derived for all-cause mortality (excluding unnatural causes of death, such as murder or accidents) rather than only mortality specifically caused by direct heat exposure. The method therefore refers to a broader mortality and temperature association than using exclusively death cases caused from direct heat impact. A denser coverage of MMTs in the global south and warmer regions would possibly have increased precision of the model by decreasing the RMSE, even though the model performed equally well for data-rich regions across different climate zones.

Current Method

Coefficents

Supplementary Table S3. Coefficients for the newly calibrated model, historic situation and new input data. Coefficients can be employed for historic and future gridded climate data as described in the Methods section in the article.

Mode	ENSMEAN
Time	2000
c	64.7446
d	0.545534305
**30-year Tmean**	0.672891536
**30-year Amplitude**	0.408054743
**Elevation**	0.006266229
**GDP/capita**	0.053298229
**Water access** (%)	-0.038787511
**RMSE**	3.2070

References

1. Riahi, K. *et al.* The Shared Socioeconomic Pathways and their energy, land use, and greenhouse gas emissions implications: An overview. *Glob. Environ. Chang.* **42**, 153–168, DOI: 10.1016/j.gloenvcha.2016.05.009 (2017).

2. Krummenauer, L. *et al.* Global drivers of minimum mortality temperatures in cities. *Sci. The Total. Environ.* **695**, 133560, DOI: 10.1016/j.scitotenv.2019.07.366 (2019).
